# Confirmatory factor analysis and Rasch analysis of the German revised version of the Niigata PPPD questionnaire: NPQ-R

**DOI:** 10.3389/fneur.2026.1797481

**Published:** 2026-04-08

**Authors:** Eve-Yaël Gerber, Roger Hilfiker, Jasmin Wandel, Frank Behrendt, Sarah Chételat, Sarah El Khadlaoui, Stefan Schädler, Maximilian Maywald, Andreas Zwergal, Leo Bonati, Sandra Becker-Bense, Corina Schuster-Amft

**Affiliations:** 1 Department of Research, Reha Rheinfelden, Rheinfelden, Switzerland; 2 Private Physiotherapy Practice Tschopp & Hilfiker, Brig, Switzerland; 3 Institute of Higher Education and Research in Healthcare, Lausanne University Hospital, Lausanne, Switzerland; 4 Institute for Optimisation and Data Analysis, Bern University of Applied Sciences, Burgdorf, Switzerland; 5 School of Engineering and Computer Science, Bern University of Applied Sciences, Biel, Switzerland; 6 School of Health Professions, Institute of Physiotherapy, Zurich University of Applied Sciences, Winterthur, Switzerland; 7 German Center for Vertigo and Balance Disorders (DSGZ), LMU University Hospital, Munich, Germany; 8 Private Physiotherapy Practice, Sumiswald, Switzerland; 9 Department of Psychiatry and Psychotherapy, LMU University Hospital, Munich, Germany; 10 Department of Neurology, LMU University Hospital, Munich, Germany; 11 Department of Clinical Research, University of Basel, Basel, Switzerland; 12 Department of Neurology, Stroke Center, University Hospital Basel, Basel, Switzerland; 13 Department of Sport, Exercise, and Health, University of Basel, Basel, Switzerland

**Keywords:** confirmatory factor analysis, functional dizziness, persistent postural-perceptual dizziness, psychometric factors, Rasch analysis

## Abstract

**Introduction:**

Persistent Postural-Perceptual Dizziness (PPPD) is a chronic functional vestibular disorder exacerbated by posture, movement, or visual stimuli. Widely used dizziness questionnaires lack specificity for PPPD symptoms. The Niigata PPPD Questionnaire (NPQ) and its German-translated and revised version (NPQ-R), including two additional subscales, were developed to address this gap. Its internal consistency, convergent validity, and test–retest reliability were found to be satisfactory. The aim of the present study was to examine the NPQ-R’s structure using confirmatory factor analysis (CFA) and Rasch item analysis.

**Materials and methods:**

We analysed data from 265 (135 female) patients (50.2 ± 16.8 years, dizziness duration 46.3 ± 76.6 months) who completed the NPQ-R. CFA was conducted using the robust maximum likelihood estimator in R (lavaan), and Rasch item analysis was performed for each subscale separately.

**Results:**

CFA revealed moderate-to-high covariances between the five latent variables (range: 0.59–0.97), with all items except one demonstrating significant standardised loadings. Rasch analysis indicated acceptable item fit for most items. Person separation reliability ranged from 0.63 to 0.75 across subscales. Item 2 (Visual Stimulation) exhibited misfit.

**Discussion:**

The NPQ-R demonstrates promising psychometric properties. While the Rasch analyses support reliability and internal coherence, the CFA results suggest that the overall five-factor model may require refinement. Conceptual clarity and wording of specific items should be re-examined. However, the NPQ-R remains suitable for PPPD assessment and severity determination in clinical and research contexts, while theoretical and empirical refinement of its factor structure is recommended.

## 1 Introduction

Persistent Postural-Perceptual Dizziness (PPPD) is a chronic functional vestibular disorder that has gained increasing clinical and scientific significance, as it is the most common chronic vestibular disorder in people aged 30 to 50 years (1, 2) and one of the most common forms of vertigo (3, 4). In 2017, the consensus committee of the Bárány Society defined PPPD as a persistent feeling of dizziness, unsteadiness, and non-spinning vertigo lasting at least for 3 months, with symptoms exacerbated by upright posture, self-or environment-induced motion, and exposure to complex visual stimuli (3). PPPD can develop either without (primary PPPD) or following an acute vestibular syndrome (secondary PPPD), such as an acute unilateral vestibulopathy, benign paroxysmal positional vertigo or vestibular migraine, or a period of heightened anxiety or psychological stress (3, 5, 6). PPPD is assumed to arise from maladaptive top-down control, characterized by a high-risk balance strategy and visual dependence (3, 7). Functional MRI studies have identified alterations in brain structure, function, and connectivity in patients with PPPD, including reduced activity of the vestibular multisensory networks and overactivity of the visual areas and other changes (8–10). The clinical presentation of PPPD is often complex and highly individual. Patients may have difficulty describing their symptoms precisely, which can contribute to underdiagnosis or misdiagnosis, particularly in primary care settings (4). Patients may use words like “lightness in the head” or “cloudiness” to explain their symptoms (3, 11). Additionally, PPPD is frequently associated with comorbid affective and anxiety disorders, including panic disorder and generalised anxiety disorder, which may predate or follow the onset of dizziness symptoms (5, 12). Notably, patients often get into a vicious cycle of emotional distress amplifying dizziness, which in turn increases anxiety and avoidance behaviour. This pattern is similar to that observed in functional neurological disorders and chronic pain conditions (5, 13).

Historically, instruments such as the Dizziness Handicap Inventory (DHI) (14, 15), the Vertigo Symptom Scale (VSS) (16, 17), and the Activities-specific Balance Confidence (ABC) Scale (18, 19) have been used to assess dizziness-related symptoms and impairment in patients with organic vestibular syndromes. While these tools provide valuable insights into the impact of dizziness on quality of life in these patient groups, they were not designed to capture the symptom profile specific to PPPD. As a result, their content validity for this disorder is limited (13, 20). To address this gap, Yagi et al. (21) developed the Niigata PPPD Questionnaire (NPQ), a Japanese self-report instrument based directly on the Bárány Society criteria. The NPQ assesses the severity of PPPD symptoms across various typical contexts, including visual stimulation, movement, and upright posture. As it was originally developed to assist in the clinical diagnosis of PPPD and to quantify symptom severity in a patient-reported format, its primary purpose is to capture the overall symptom profile and severity associated with PPPD rather than to differentiate between potential clinical subtypes of the disorder. In 2025, the Athens–Lübeck Questionnaire (ALQ) was introduced as an instrument specifically designed to assess potential PPPD subtypes (22). Accordingly, the NPQ should primarily be understood as a tool for diagnostic support and severity assessment. Its utility has since been demonstrated in both Japanese and Western populations (23, 24), and it has been adapted into several languages, including French (24), Spanish (23) and German (25). The German revised version of the instrument (NPQ-R) was developed through cognitive debriefing interviews with PPPD patients and an expert Delphi consensus process to enhance content validity and clinical relevance (26). This version introduced additional items and a more refined item structure to better reflect patients’ experiences. In a recent publication, Chételat et al. (25) presented the first German translations and validations of both the original NPQ and the NPQ-R, following internationally accepted guidelines for cross-cultural adaptation (27, 28). The study confirmed the internal consistency, convergent validity, and test–retest reliability of both instruments in a German-speaking clinical sample.

The availability of the culturally adapted and psychometrically validated German NPQ-R represents a major step forward for clinical and research applications. However, further psychometric evaluation is needed to confirm the underlying factor structure and assess model fit in a larger, more diverse sample. Thus, the aim of the present study was to examine confirmatory factor analysis and Rasch item analysis of the German revised version of the Niigata PPPD Questionnaire (NPQ-R) to support diagnostic procedures and monitoring of treatment in PPPD patients. We hypothesised confirmation of the five-factor structure, comprising the three original factors proposed by Yagi et al. (21) and the two additional factors introduced in the German revised version of the questionnaire by Behrendt et al. (26).

## 2 Materials and methods

### 2.1 Study design, recruitment, and study patients

This study is a continuation of the German adaptation and validation of the original and revised Niigata PPPD Questionnaire (NPQ and NPQ-R) conducted by Chételat et al. (25) and Behrendt et al. (26). As part of a larger project, the current work aimed to evaluate the factorial structure of the revised German NPQ-R in a sample of German-speaking patients with PPPD using confirmatory factor analysis. The five-factor model tested was based on both theoretical foundations and previous empirical findings (26).

This study included data from 265 patients with PPPD in Switzerland and Germany, recruited between September 2021 and December 2023. In Switzerland, patients were recruited at the Cantonal Hospitals of Lucerne, the University Hospitals of Zurich and Basel, Reha Rheinfelden, and various private practices. In Germany, all patients were recruited at the outpatient unit of the German Center for Vertigo and Balance Disorders (DSGZ) at the LMU University Hospital in Munich. Out of the 265 included patients, 140 (52.8%) were diagnosed with primary and 125 (47.2%) with secondary PPPD.

Patient inclusion criteria were a diagnosis of PPPD, age ≥ 18 years, good German language proficiency, and signed informed consent (25). Exclusion criteria were other forms of vestibular disorders as defined by the International Classification of Vestibular Disorders (ICVD). Detailed information on patient recruitment, diagnostics, and study methodology can be found in the recently published open access article by Chételat et al. (25).

### 2.2 Measures: NPQ-R

The NPQ-R is a 19-item self-report instrument developed to assess the core symptom dimensions of PPPD, whereas the subscales were defined as “Upright Posture,” “Movement,” “Visual Stimulation,” “Associated Symptoms” and “Symptom Behaviour” (26). Items are rated on a seven-point Likert scale ranging from 0 (“I have no complaints”/“Does not apply at all”) to 6 (“Is it unbearable”/“Fully applies”), with higher scores indicating greater symptom severity. Maximum total score of the NPQ-R is 114 points. The German NPQ-R was developed following cross-cultural adaptation guidelines by Beaton et al., 2000 (26) and validated for internal consistency, convergent validity, and test–retest reliability (25).

### 2.3 Statistical analysis

The dataset was compiled in Microsoft Excel and imported into R (Version 2025.05.1 + 513) for statistical analysis. The R packages lavaan, laavanPlot, dplyr, openxlsx, DiagrammeR and eRm were used for analysis. Level of significance was set at *p* ≤ 0.05.

As described in Chételat et al. (25), questionnaires with missing values exceeding 15% were excluded from analysis to mitigate potential selection bias (29). Therefore, two questionnaires containing more than 15% missing data were excluded from the analysis and the remaining questionnaires (≤15% missing data) were retained and analysed using full information maximum likelihood estimation. Demographics and test data were analysed using descriptive statistics in the preceding study conducted by Chételat et al. (25). Floor or ceiling effects are present when 15% of subjects achieve the highest or lowest possible score (30).

Data were screened for univariate normality by calculating skewness and kurtosis for each item. Items with skewness between −2 and +2 and kurtosis between −7 and +7 were considered approximately normal (31). These criteria were supported by visual inspection using histograms and Q-Q plots.

The confirmatory factor analysis was performed to test the previously proposed five-factor model of the NPQ-R, in which the 19 items were assigned to the following latent variables: (1) Upright Posture (Items: 4, 11, 12, 18), (2) Movement (Items: 1, 8, 15, 19), (3) Visual Stimulation (Items: 2, 6, 13, 16), (4) Associated Symptoms (Items: 3, 5, 9, 17), and (5) Symptom Behaviour (Items: 7, 10, 14). Due to deviations from normality in some items, the robust maximum likelihood estimator was used in lavaan to estimate model parameters. Missing data were handled using full information maximum likelihood, as implemented in the package.

The following fit indices were evaluated to assess model adequacy: comparative fit index: ≥ 0.95 (acceptable), ≥ 0.97 (good); Tucker–Lewis index: ≥ 0.90 (acceptable), ≥ 0.95 (excellent); root mean square error of approximation: ≤ 0.06; standardised root mean square residual: ≤ 0.08 (32).

Chi-square statistics and degrees of freedom were also computed. Fit indices were extracted using the fitMeasures() function, and parameter estimates (standardised and unstandardised) were obtained via the parameterEstimates() function in lavaan.

To further investigate the item properties of the NPQ-R, a Rasch analysis was conducted using the partial credit model for each of the five subscales individually. The Rasch analysis is a psychometric method designed to enhance the development, evaluation and refinement of psychological assessment instruments (33, 34). The focus was on evaluating item fit using outfit and infit mean square statistics. Reasonable item mean square ranges for infit and outfit was defined as 0.6–1.4 (35). Further, the person separation reliability was computed for each subscale. Associated t-statistics were not used due to their sensitivity to sample size. Given the size of our sample, t-statistics are liable to identify trivial misfit as statistically significant.

## 3 Results

### 3.1 Study sample

The demographic characteristics reported in this study are based on the analyses originally conducted by Chételat et al. (25) and were adopted here as a secondary analysis. The sample included 265 patients (mean age 50.2 years, SD 16.8; 130 males: 47.1, SD 17.2; 135 females: 53.1, SD 15.8; dizziness duration 46.3 months, SD 76.6). In total, 117 patients were recruited from different centres in Switzerland, and 148 patients were recruited from the DSGZ at the Ludwig-Maximilians-University Munich. The average duration of dizziness was 46.3 (SD: 76.6) months. For more detailed information regarding sex-specific analysis, please refer to Chételat et al. (25).

### 3.2 Confirmatory factor analysis

**Figure 1:**
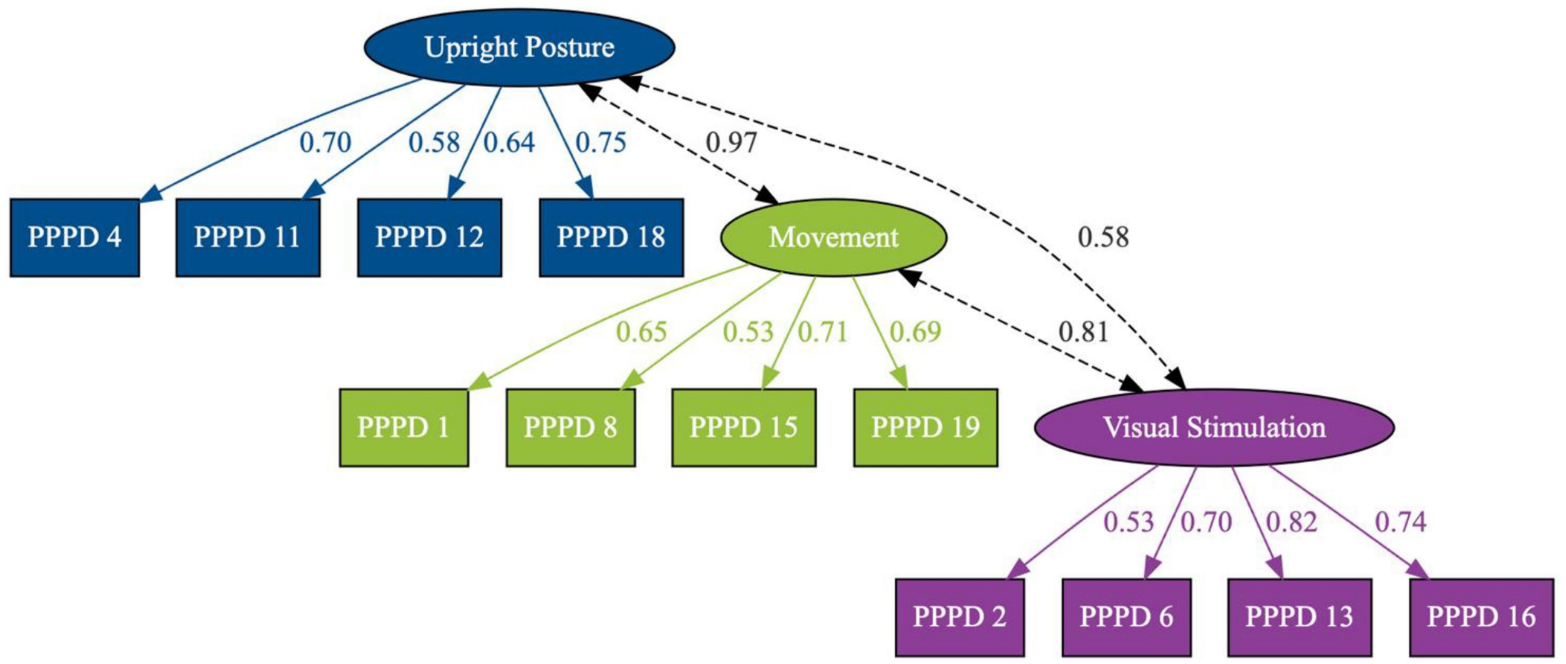
Path diagram of the confirmatory factor analysis model of the NPQ (N = 265). The figure the standardised factor loadings (outer paths) and standardised latent covariances (curved double-headed arrows) for each subscale. NPQ, Niigata PPPD Questionnaire (12-items).

For the confirmatory factor analysis model estimation, data from all 265 patients were used. All 19 NPQ-R items were included in the analysis. Analysis of the NPQ (12-items) can be found as Table 1 and Figure 1. Descriptive checks showed that skewness values for the items ranged from −1.02 (PPPD_17) to 0.57 (PPPD_16), and kurtosis values ranged from 1.84 (PPPD_6) to 3.42 (PPPD_17). Histograms and Q–Q plots indicated minor deviations from normality. Based on these results, model estimation was performed using the robust maximum likelihood estimator with full information maximum likelihood for missing data.

**Table 1:** Confirmatory factor analysis factor loadings for the NPQ (*N* = 265).

Subscale	Item	Item question	Estimate	Standard error	*z*-value	*P*(>|z|)	Standardized factor loading
Upright Posture	PPPD_4	Wenn ich in meinem eigenen Tempo zu Fuss gehe, dann … *When I walk at my own pace, then …*	1	0			0.70
	PPPD_11	Wenn ich länger auf einem Hocker oder einem Stuhl ohne Rücken- oder Armlehnen sitze, dann … *If I sit for a longer period of time on a stool or a chair without back or armrests, then …*	0.96	0.15	6.49	< 0.001	0.58
	PPPD_12	Wenn ich länger frei stehe ohne mich fest zu halten oder mich auf zu stützen, dann … *If I stand freely for a longer period of time without holding on or leaning up, then …*	1.13	0.14	7.85	< 0.001	0.64
	PPPD_18	Wenn ich mit grossen Schritten und eher schnell gehe, dann … *When I walk with big steps and rather fast, then …*	1.14	0.09	12.21	< 0.001	0.75
Movement	PPPD_1	Wenn ich schnell aufstehe, mich schnell umdrehe oder bei ähnlichen Bewegungen, dann … *When I stand up quickly, turn around quickly, or in similar movements, then …*	1	0			0.65
	PPPD_8	Wenn ich mit Auto, Bus, Zug oder anderen Verkehrsmitteln fahre, dann … *When I travel by car, bus, train or other means of transport, then …*	0.88	0.12	7.03	< 0.001	0.53
	PPPD_15	Wenn ich mich im Haushalt oder bei leichtem Sport bewege, dann … *When I am doing household chores or light sports, then …*	1.04	0.1	9.96	< 0.001	0.71
	PPPD_19	Wenn ich Rolltreppen oder einen Aufzug benutze, dann … *When I use escalators or an elevator, then …*	1.16	0.12	9.35	< 0.001	0.69
Visual Stimulation	PPPD_2	Wenn ich Regale im Supermarkt oder Baumarkt durchsehe, dann … *When I look through shelves in the supermarket or hardware store, then …*	1	0			0.53
	PPPD_6	Wenn ich in Film oder Fernsehen schnelle/hektische Bilder sehe, dann … *When I see fast/hectic images in movies or on TV, then …*	1.48	0.25	5.98	< 0.001	0.70
	PPPD_13	Wenn ich auf einem PC oder Smartphone den Bildschirminhalt durchscrolle, dann … *When I scroll through the screen contents on a PC or smartphone, then …*	1.62	0.27	6	< 0.001	0.82
	PPPD_16	Wenn ich klein gedruckte Schrift in Büchern oder der Zeitung lese, dann … *When I read small print in books or the newspaper, then …*	1.31	0.19	6.77	< 0.001	0.74

The table presents the factor loadings for each item across the three latent subscales. Estimates were obtained using robust maximum likelihood (MLR) with full information maximum likelihood for missing data. NPQ, Niigata PPPD Questionnaire (12-items).

Standardised covariances between latent variables ranged from 0.59 to 0.97. The covariance between Upright Posture and Movement was 0.97. Other covariances were:

- Upright Posture – Visual Stimulation = 0.59,
- Upright Posture – Associated Symptoms = 0.71,
- Upright Posture – Symptom Behaviour = 0.96,
- Movement – Visual Stimulation = 0.82,
- Movement – Associated Symptoms = 0.75,
- Movement – Symptom Behaviour = 0.96,
- Visual Stimulation – Associated Symptoms = 0.66,
- Visual Stimulation – Symptom Behaviour = 0.84, and
- Associated Symptoms – Symptom Behaviour = 0.89.

Model fit indices were as follows: *χ*^2^ = 563.90, degrees of freedom = 142, *p <* 0.001; comparative fit index = 0.80; Tucker-Lewis index = 0.77; root mean square error of approximation = 0.11; standardised root mean square residual = 0.08.

All items showed significant standardised factor loadings (all *p <* 0.001) except PPPD_9 (*p* = 0.07). The standardised factor loadings can be seen in Table 2.

**Table 2:** Confirmatory factor analysis factor loadings for the NPQ-R (N = 265).

Subscale	Item	Item question	Estimate	Standard error	*z*-value	P(>|z|)	Standardized factor loading
Upright Posture	PPPD_4	Wenn ich in meinem eigenen Tempo zu Fuss gehe, dann … *When I walk at my own pace, then …*	1	0			0.70
	PPPD_11	Wenn ich länger auf einem Hocker oder einem Stuhl ohne Rücken- oder Armlehnen sitze, dann … *If I sit for a longer period of time on a stool or a chair without back or armrests, then …*	1.00	0.14	7.25	< 0.001	0.60
	PPPD_12	Wenn ich länger frei stehe ohne mich fest zu halten oder mich auf zu stützen, dann … *If I stand freely for a longer period of time without holding on or leaning up, then …*	1.18	0.14	8.65	< 0.001	0.67
	PPPD_18	Wenn ich mit grossen Schritten und eher schnell gehe, dann … *When I walk with big steps and rather fast, then …*	1.10	0.09	11.83	< 0.001	0.71
Movement	PPPD_1	Wenn ich schnell aufstehe, mich schnell umdrehe oder bei ähnlichen Bewegungen, dann … *When I stand up quickly, turn around quickly, or in similar movements, then …*	1	0			0.64
	PPPD_8	Wenn ich mit Auto, Bus, Zug oder anderen Verkehrsmitteln fahre, dann … *When I travel by car, bus, train or other means of transport, then …*	0.92	0.13	6.96	< 0.001	0.55
	PPPD_15	Wenn ich mich im Haushalt oder bei leichtem Sport bewege, dann … *When I am doing household chores or light sports, then …*	1.05	0.11	9.68	< 0.001	0.70
	PPPD_19	Wenn ich Rolltreppen oder einen Aufzug benutze, dann … *When I use escalators or an elevator, then …*	1.18	0.13	9.09	< 0.001	0.69
Visual Stimulation	PPPD_2	Wenn ich Regale im Supermarkt oder Baumarkt durchsehe, dann … *When I look through shelves in the supermarket or hardware store, then …*	1	0			0.55
	PPPD_6	Wenn ich in Film oder Fernsehen schnelle/hektische Bilder sehe, dann … *When I see fast/hectic images in movies or on TV, then …*	1.43	0.23	6.15	< 0.001	0.70
	PPPD_13	Wenn ich auf einem PC oder Smartphone den Bildschirminhalt durchscrolle, dann … *When I scroll through the screen contents on a PC or smartphone, then …*	1.54	0.25	6.12	< 0.001	0.81
	PPPD_16	Wenn ich klein gedruckte Schrift in Büchern oder der Zeitung lese, dann … *When I read small print in books or the newspaper, then …*	1.26	0.19	6.72	< 0.001	0.73
Associated Symptoms	PPPD_3	Wenn ich Schwindel habe, dann habe ich Mühe, mich zu konzentrieren. *When I have dizziness, I have trouble concentrating.*	1	0			0.62
	PPPD_5	Wenn ich Schwindel habe, dann fühle ich mich verängstigt oder verunsichert. *When I have dizziness, I feel scared or anxious.*	1.19	0.28	4.29	< 0.001	0.65
	PPPD_9	Wenn ich zu Fuss gehe, dann fühle ich mich unsicher. *When I walk, I feel insecure.*	1.02	0.56	1.81	0.07	0.56
	PPPD_17	Wenn ich Schwindel habe, dann ist meine Leistungsfähigkeit eingeschränkt. *When I have dizziness, my performance is limited (*e.g.*, at work, with childcare, with domestic activities).*	1.08	0.13	8.57	< 0.001	0.69
Symptom Behaviour	PPPD_7	Wenn ich mir eine Pause gönne oder mich ausruhe, dann … *When I give myself a break or rest, then …*	1	0			0.49
	PPPD_10	Wenn ich mich in unruhiger Umgebung befinde (z. B. Menschenmenge, Verkehr), dann … *When I am in an unsettled environment (*e.g.*, crowd, traffic), then …*	1.68	0.23	7.38	< 0.001	0.68
	PPPD_14	Wenn ich mich ablenke z. B. durch eine Tätigkeit oder meine Gedanken auf etwas anderes richte, dann … *If I distract myself,* e.g.*, by an activity or direct my thoughts on something else, then …*	1.17	0.14	8.14	< 0.001	0.60

The table presents the factor loadings for each item across the five latent subscales. Estimates were obtained using robust maximum likelihood (MLR) with full information maximum likelihood for missing data. NPQ-R, Niigata PPPD Questionnaire Revised version (19-items).

Intercepts for the observed items ranged from 1.73 (PPPD_7) to 4.46 (PPPD_17). Standardised residual variances ranged from 0.34 (PPPD_13) to 0.76 (PPPD_7). All item residual variances were significant (*p <* 0.05). Latent variable variances were Upright Posture = 1.01 (*p <* 0.00), Movement = 0.99 (*p <* 0.00), Visual Stimulation = 0.89 (*p <* 0.00), Associated Symptoms = 0.97 (*p* = 0.05), and Symptom Behaviour = 0.47 (*p <* 0.00).

**Figure 2:**
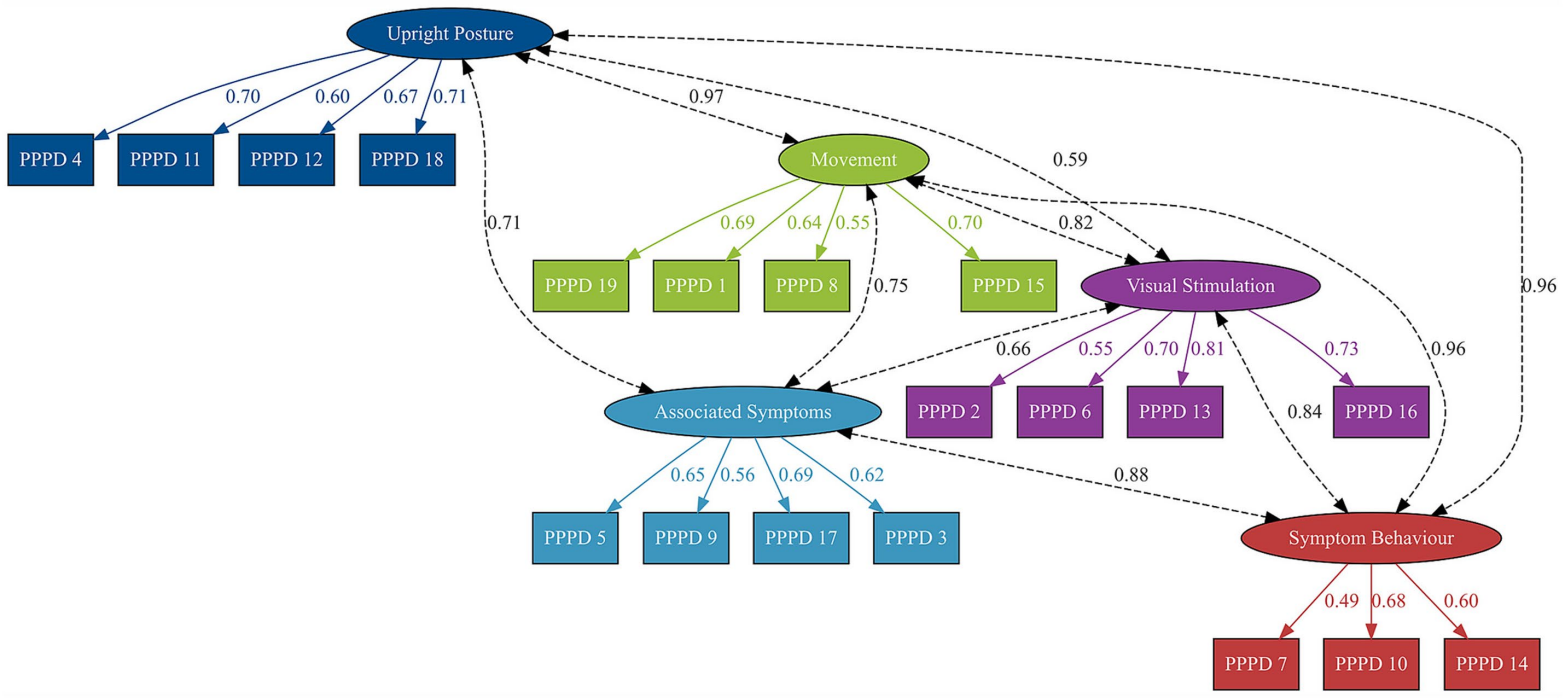
Path diagram of the confirmatory factor analysis model of the NPQ-R. Legend: The figure shows the standardised factor loadings (outer paths) and standardised latent covariances (curved double-headed arrows) for each subscale. NPQ-R, Niigata PPPD Questionnaire Revised version (19-items).

A graphical illustration of the confirmatory factor analysis, including standardised factor loadings and standardised covariances between latent variables, is provided in Figure 2.

### 3.3 Rasch analysis

For the Upright Posture subscale (PPPD_4, PPPD_11, PPPD_12, PPPD_18), outfit mean square statistic values ranged from 0.74 to 0.92, and infit mean square statistic values from 0.72 to 0.90. Person separation reliability was 0.75.

In the Movement subscale (PPPD_1, PPPD_8, PPPD_15, PPPD_19), outfit mean square statistics ranged from 0.69 to 0.93, and infit mean square statistics from 0.70 to 0.92. Person separation reliability was 0.73.

For the Visual Stimulation subscale (PPPD_2, PPPD_6, PPPD_13, PPPD_16), outfit mean square statistics ranged from 0.62 to 1.18 and infit mean square statistics from 0.62 to 1.13. One item (PPPD_2) had elevated outfit and infit mean square statistic values (1.18 and 1.13, respectively). Person separation reliability was 0.72.

The Associated Symptoms subscale (PPPD_3, PPPD_5, PPPD_9, PPPD_17) presented outfit mean square statistics from 0.64 to 1.05 and infit mean square statistics from 0.65 to 1.06. PPPD_9 was the only item with mean square statistics slightly above 1.0. Person separation reliability was 0.70.

In the Symptom Behaviour subscale (PPPD_7, PPPD_10, PPPD_14), outfit mean square statistics ranged from 0.67 to 0.78 and infit mean square statistics from 0.68 to 0.76. Person separation reliability was 0.63.

**Figure 3:**
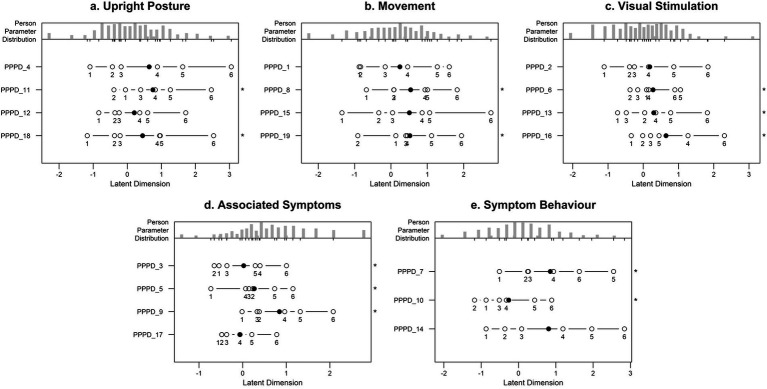
Wright maps. The figure shows the Wright maps of the Rasch analysis for each subscale of the NPQ-R **(a)** Upright posture; **(b)** Movement; **(c)** Visual Stimulation; **(d)** Associated Symptoms; **(e)** Symptom Behaviour.

Person separation reliability and item fit statistics for all subscales of the NPQ-R are summarised in Table 3. Wright maps are presented in Figure 3.

**Table 3:** Person separation reliability and item fit statistics from the Rasch analysis of the NPQ-R.

Subscale	Person separation reliability	Item	Item question	Outfit mean square statistics	Infit mean square statistics
Upright Posture	0.75	PPPD_4	Wenn ich in meinem eigenen Tempo zu Fuss gehe, dann … *When I walk at my own pace, then …*	0.74	0.75
		PPPD_11	Wenn ich länger auf einem Hocker oder einem Stuhl ohne Rücken- oder Armlehnen sitze, dann … *If I sit for a longer period of time on a stool or a chair without back or armrests, then …*	0.92	0.90
		PPPD_12	Wenn ich länger frei stehe ohne mich fest zu halten oder mich auf zu stützen, dann … *If I stand freely for a longer period of time without holding on or leaning up, then …*	0.75	0.72
		PPPD_18	Wenn ich mit grossen Schritten und eher schnell gehe, dann … *When I walk with big steps and rather fast, then …*	0.80	0.79
Movement	0.73	PPPD_1	Wenn ich schnell aufstehe, mich schnell umdrehe oder bei ähnlichen Bewegungen, dann … *When I stand up quickly, turn around quickly, or in similar movements, then …*	0.84	0.83
		PPPD_8	Wenn ich mit Auto, Bus, Zug oder anderen Verkehrsmitteln fahre, dann … *When I travel by car, bus, train or other means of transport, then …*	0.93	0.92
		PPPD_15	Wenn ich mich im Haushalt oder bei leichtem Sport bewege, dann … *When I am doing household chores or light sports, then …*	0.72	0.72
		PPPD_19	Wenn ich Rolltreppen oder einen Aufzug benutze, dann … *When I use escalators or an elevator, then …*	0.69	0.70
Visual Stimulation	0.72	PPPD_2	Wenn ich Regale im Supermarkt oder Baumarkt durchsehe, dann … *When I look through shelves in the supermarket or hardware store, then …*	1.18	1.13
		PPPD_6	Wenn ich in Film oder Fernsehen schnelle/hektische Bilder sehe, dann … *When I see fast/hectic images in movies or on TV, then …*	0.72	0.77
		PPPD_13	Wenn ich auf einem PC oder Smartphone den Bildschirminhalt durchscrolle, dann … *When I scroll through the screen contents on a PC or smartphone, then …*	0.62	0.62
		PPPD_16	Wenn ich klein gedruckte Schrift in Büchern oder der Zeitung lese, dann … *When I read small print in books or the newspaper, then …*	0.65	0.65
Associated Symptoms	0.70	PPPD_3	Wenn ich Schwindel habe, dann habe ich Mühe, mich zu konzentrieren. *When I have dizziness, I have trouble concentrating.*	0.78	0.74
		PPPD_5	Wenn ich Schwindel habe, dann fühle ich mich verängstigt oder verunsichert. *When I have dizziness, I feel scared or anxious.*	0.64	0.65
		PPPD_9	Wenn ich zu Fuss gehe, dann fühle ich mich unsicher. *When I walk, I feel insecure.*	1.05	1.06
		PPPD_17	Wenn ich Schwindel habe, dann ist meine Leistungsfähigkeit eingeschränkt. *When I have dizziness, my performance is limited (*e.g.*, at work, with childcare, with domestic activities).*	0.67	0.69
Symptom Behaviour	0.63	PPPD_7	Wenn ich mir eine Pause gönne oder mich ausruhe, dann … *When I give myself a break or rest, then …*	0.78	0.76
		PPPD_10	Wenn ich mich in unruhiger Umgebung befinde (z. B. Menschenmenge, Verkehr), dann … *When I am in an unsettled environment (*e.g.*, crowd, traffic), then …*	0.67	0.68
		PPPD_14	Wenn ich mich ablenke z. B. durch eine Tätigkeit oder meine Gedanken auf etwas anderes richte, dann … *If I distract myself,* e.g.*, by an activity or direct my thoughts on something else, then …*	0.72	0.69

The table shows the person separation reliability and item fit statistics from the Rasch analysis. Rasch analysis was performed using the partial credit model separately for each NPQ-R subscale. NPQ-R, Niigata PPPD Questionnaire Revised version (19-items).

## 4 Discussion

Building on the German adaptation and initial validation presented by Chételat et al. (25), the present study aimed to evaluate the factorial validity of the German NPQ-R using confirmatory factor analysis and to explore its psychometric performance using Rasch analysis.

### 4.1 Confirmatory factor analysis

The five-factor model of the NPQ-R, containing the five subscales, was tested using robust maximum likelihood estimation in a sample of 265 German-speaking PPPD patients. Preliminary inspection of the data revealed minor deviations from univariate normality, and the use of the robust maximum likelihood estimator was therefore deemed appropriate. The confirmatory factor analysis results indicated that all items except PPPD_9 (Associated Symptoms: “When I walk, I feel insecure.”) showed statistically significant factor loadings on their intended latent dimensions, suggesting an overall good fit of items within their respective subscales and a limited contribution of PPPD_9 to its underlying subscale. While the statistical performance of PPPD_9 is limited, it captures a highly relevant aspect of the disorder in daily life. Walking is a situation that patients frequently encounter in their everyday activities and is therefore considered clinically important for assessing PPPD symptomatology. Most standardised factor loadings were within an acceptable range, supporting the internal consistency of the five subscales.

However, the significant factor loadings on their intended latent dimensions should be interpreted with caution, as model fit indices were below commonly accepted thresholds for good model fit. Specifically, both the comparative fit index (0.80) and the Tucker–Lewis index (0.77) fell below the recommended cutoff of 0.90 (32), and the root mean square error of approximation (0.11) exceeded the maximum acceptable value of 0.06 (32). Additionally, a warning was generated during estimation indicating that the latent covariance matrix was not positive definite. Inspection of the latent factor covariances revealed a very high correlation between the Upright Posture and Movement subscales (r = 0.97), suggesting limited discriminant validity between these two dimensions and indicating that the two factors may not represent clearly distinguishable constructs. It further highlights the need for caution when interpreting the distinctiveness of the NPQ-R subscales. These results suggest that, while the five-factor structure broadly reflects the theoretical model underpinning the NPQ-R, the data from this sample show substantial overlap among some constructs. This may be partly attributable to the symptomatology of PPPD, in which postural and movement-related discomfort often co-occur and are not clearly distinguished by patients. Furthermore, inadequate musculoskeletal stiffening with co-contraction of antigravity muscles may contribute to this pattern by simultaneously affecting stance and gait (36, 37). In this context, the five-factor structure may be best understood as a theoretically informed framework. In particular, the first three subscales were adapted from the original structure proposed by Yagi et al. (21), while the additional subscales were introduced in later work by Behrendt et al. (26) to capture further clinically relevant aspects of PPPD symptomatology. Importantly, the NPQ was primarily designed to support the diagnosis of PPPD and to assess the severity of its core symptoms rather than to differentiate between potential clinical subtypes of the disorder. Finally, the comparatively high residual variances observed for some items and the lower variance of the Symptom Behaviour subscale suggest that certain aspects of PPPD symptoms may not be fully captured by the current factor structure, which may further contribute to the limited global model fit and the strong correlations between some latent dimensions.

### 4.2 Rasch analysis

The Rasch analysis was used to provide an additional insight into item functioning. Across subscales, most items demonstrated outfit and infit mean square statistic values within the acceptable range (0.6–1.4) (35) indicating an overall acceptable model fit.

The Upright Posture and Movement subscales showed the highest person separation reliability (0.75 and 0.73, respectively), suggesting satisfactory ability to distinguish among different levels of symptom severity. Symptom Behaviour, which included only three items, demonstrated the lowest reliability (0.63), which could reflect insufficient item coverage or a narrower construct.

PPPD_2 within the Visual Stimulation subscale showed elevated misfit values, and item PPPD_9 in the Associated Symptoms subscale displayed weak item fit across all items. These results are consistent with the confirmatory factor analysis findings and suggest to re-examine the conceptual clarity and wording of these items. Still, the Visual Stimulation and Associated Symptoms subscales yielded adequate reliability (0.72 and 0.70), suggesting that the subscales may still provide useful information despite isolated item-level concerns.

### 4.3 Strengths and limitations

A limitation of this study lies in the high inter-factor correlations observed in the confirmatory factor analysis, particularly between the factors Upright Posture and Movement, which may challenge the discriminant validity of these constructs. At the same time, the study’s large sample size, the largest reported to date for a validation of this instrument, enhances the reliability of the statistical results. Significant item-level results should be interpreted alongside effect sizes and overall model fit when evaluating the measurement structure, as in larger samples statistical significance may be achieved even for relatively small effects. Unlike the original Japanese (21), Spanish (23), and French (24) versions, the present study additionally conducted both an exploratory factor analysis and a Rasch analysis, providing a more detailed examination of the psychometric properties for the German NPQ-R. Another limitation is the poor fit of individual items in the Rasch model, such as PPPD_9 and PPPD_2, which could affect the precision of subscale scores. However, the inclusion of participants from two countries, different clinical care institutions (e.g., outpatient, stationary, rehabilitative, tertiary care), and the nearly equal gender distribution strengthen the generalisability of the findings.

## 5 Conclusion and outlook

PPPD requires comprehensive assessment tools that reflect both the sensory and cognitive-emotional dimensions of the disorder. The NPQ-R, developed to address this need and to operationalise the current diagnostic criteria proposed by the Bárány Society in the form of a standardised patient-reported outcome measure, demonstrates overall good structural validity and acceptable psychometric performance. However, certain subscales and items may require revision or more precise definition. To address the high inter-factor correlations identified in the confirmatory factor analysis, specifically between the subscales Upright Posture and Movement, future studies could explore alternative structural models, which might better capture the underlying dimensionality. Further, revision or replacement of poorly performing items like PPPD_9 and PPPD_2 may help improve psychometric properties. Lastly, validation studies linking NPQ-R subscales to clinical outcomes, treatment responses, and cross-cultural measurement invariance are needed to support the instrument’s broader clinical and research utility.

## Data Availability

The raw data supporting the conclusions of this article will be made available by the authors, without undue reservation.
